# Two-Year Outcomes and Interictal Burden After Treatment for Medication Overuse Headache

**DOI:** 10.3390/jcm15124785

**Published:** 2026-06-19

**Authors:** Yooha Hong, Mi-Kyoung Kang, Soo-Jin Cho

**Affiliations:** Department of Neurology, Dongtan Sacred Heart Hospital, Hallym University College of Medicine, 7, Keunjaebong-gil, Hwaseong 18450, Gyeonggi-do, Republic of Korea; dbgk486@naver.com (Y.H.); alroddlbebe@gmail.com (M.-K.K.)

**Keywords:** medication overuse headache, interictal burden, migraine, migraine interictal burden scale, quality of life

## Abstract

**Background/Objective:** Medication overuse headache (MOH) is a disabling secondary headache disorder that arises from an underlying primary headache, most commonly migraine. Although treatment reduces headache frequency and medication overuse, the interictal burden—the impact experienced between headache attacks—remains poorly characterized over the long term. This study evaluated interictal burden and clinical outcomes two years after MOH diagnosis. **Methods:** This study was derived from a prospective multicenter cohort of patients with MOH, using data from a single center. Of 149 patients enrolled between April 2020 and November 2022, 117 (78.5%) completed the two-year follow-up. Clinical characteristics, medication overuse, monthly headache days, and standardized questionnaires were assessed at baseline and follow-up. Interictal burden was evaluated at two years using the Migraine Interictal Burden Scale (MIBS-4), with scores ≥5 indicating severe burden. **Results:** At baseline, patients (81.2% female; median age, 45.0 years) reported a median of 16.0 monthly medication days (interquartile range, 13.0–23.0). Medication overuse decreased from 100% at baseline to 24.2% at one year and 17.1% at two years. Among 117 patients with available two-year MIBS-4 data, 25 (21.4%) had severe interictal burden. Compared with those without severe burden, these patients had greater headache-related impact and disability (HIT-6: 68.0 vs. 64.0, *p* = 0.019; MIDAS: 110.0 vs. 36.0, *p* = 0.002), higher psychological burden (PHQ-9: 11.0 vs. 8.0, *p* = 0.032; GAD-7: 7.0 vs. 4.0, *p* = 0.010), and were more likely to be current smokers (20.0% vs. 4.3%, *p* = 0.036). Notably, 14.4% of patients with resolved medication overuse still reported severe interictal burden. **Conclusions:** Two years after MOH diagnosis, severe interictal burden was observed in a substantial proportion of patients and was associated with greater baseline disability and psychological distress. These findings highlight the need for long-term monitoring and management beyond initial medication withdrawal.

## 1. Introduction

Medication overuse headache (MOH) is a secondary headache disorder that develops in individuals with an underlying primary headache, most commonly migraine or tension-type headache, due to the regular overuse of acute symptomatic medications. It is characterized by a chronic headache pattern occurring on ≥15 days per month and is associated with significant disability. MOH affects approximately 1–2% of the general population, but its prevalence is markedly higher in tertiary headache centers, where it accounts for up to 70% of referrals [1,2]. Its reported prevalence varies widely due to evolving diagnostic criteria and heterogeneous definitions across studies [3].

MOH poses a considerable burden on both patients and healthcare systems [4]. The persistent headaches and associated symptoms can disrupt daily activities, reduce productivity, and contribute to psychological distress, including anxiety and depression [5,6]. MOH also increases healthcare utilization, including medical consultations and emergency department visits, resulting in substantial direct and indirect economic costs [7]. While the impact of MOH during headache attacks has been well-documented, emerging evidence suggests that patients also experience a significant burden during the interictal phase—defined as the period between acute headache episodes [8,9]. This interictal burden includes anticipatory anxiety, limitations in planning social or leisure activities, and persistent functional impairment, even in the absence of active headache. However, most clinical studies have focused primarily on ictal metrics such as headache frequency or medication use, with limited attention to the interictal phase. Furthermore, few studies have examined long-term outcomes in MOH, and those that have are typically restricted to follow-up periods of 6 to 12 months. As a result, little is known about the trajectory of interictal burden in MOH, or the clinical and psychological factors that may contribute to its severity.

In this single-center sub-study of a prospective MOH registry, we evaluated interictal burden at two years using the validated Migraine Interictal Burden Scale (MIBS-4), while also characterizing the two-year clinical course of MOH and identifying its potential correlates. By addressing this gap, our findings may help refine long-term management strategies for MOH, with a focus not only on reducing headache frequency and medication use but also on improving quality of life between attacks.

## 2. Materials and Methods

### 2.1. Study Design and Participants

This study was based on the Registry for Load and Management of MEdicAtion OveruSE Headache [10], a nationwide multicenter prospective observational study designed to investigate the clinical characteristics and outcomes of MOH in South Korea. From this registry, 149 patients with MOH were consecutively enrolled at a single headache center between April 2020 and November 2022. While the original multicenter protocol included a one-year follow-up, patients at this center underwent an extended two-year follow-up. As systematic prospective assessment of interictal burden using the MIBS-4 through this extended two-year follow-up was available only at this center, the present analysis was conducted as a single-center sub-study of the multicenter registry. All patients met the diagnostic criteria for MOH according to the International Classification of Headache Disorders, 3rd edition (ICHD-3) [11]. Eligible participants were required to be aged ≥19 years, able to communicate and complete study questionnaires, and able to provide written informed consent. Patients were excluded if they had severe neurological, psychiatric, or systemic medical conditions that could interfere with participation or data validity.

**Figure 1:**
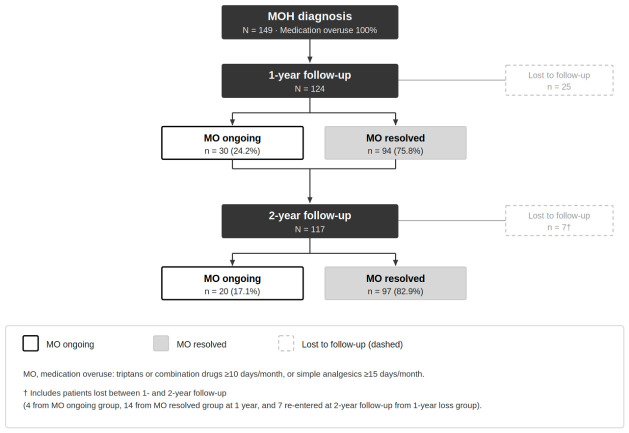
Patient selection and follow-up Among 149 patients diagnosed with medication overuse headache (MOH) at baseline, 124 (83.2%) completed the 1-year follow-up and 117 (78.5%) completed the 2-year follow-up. At 1-year follow-up, medication overuse (MO) was ongoing in 30 patients (24.2%) and resolved in 94 patients (75.8%), with 25 lost to follow-up. At 2-year follow-up, MO was ongoing in 20 patients (17.1%) and resolved in 97 patients (82.9%), with 7 additional patients lost to follow-up. Dashed boxes indicate patients lost to follow-up. Solid white boxes indicate ongoing MO; shaded boxes indicate resolved MO. Abbreviations: MOH, medication overuse headache; MO, medication overuse (triptans or combination drugs ≥10 days/month, or simple analgesics ≥15 days/month).

Of the 149 patients enrolled at baseline, 124 completed the one-year follow-up (25 lost to follow-up), and 117 patients (78.5%) completed the full two-year follow-up and were included in the final analysis (Figure 1). Within this registry, management of MOH was not protocol-driven but individualized at the discretion of the treating headache specialist, reflecting real-world clinical practice. Treatment was primarily conducted in the outpatient setting and consisted of withdrawal of the overused acute medication, either through abrupt discontinuation or gradual tapering, together with initiation or optimization of preventive therapy as clinically indicated. Preventive treatments included oral preventive medications, onabotulinumtoxinA, and CGRP monoclonal antibodies. Details of acute and preventive medication use at baseline and each follow-up visit are provided in Tables S2 and S3.

### 2.2. Clinical Assessments

At baseline, participants underwent comprehensive assessments, including collection of demographic data, headache characteristics (monthly headache days, monthly severe headache days, and monthly medication days), and completion of validated questionnaires evaluating headache impact, disability, and psychological comorbidities. Information on medication use was also collected at baseline, including both acute and preventive treatments. Acute medication classes (e.g., simple analgesics, combination analgesics, triptans, ergotamine, and opioids) and preventive medication use were recorded.

To assess the impact of headaches on daily life, we used the Headache Impact Test-6 (HIT-6), with a score of ≥60 indicating a severe impact [12]. Headache-related disability was measured using the Migraine Disability Assessment Scale (MIDAS) [13]. Psychological comorbidities were evaluated using the Patient Health Questionnaire-9 (PHQ-9) for depression and the Generalized Anxiety Disorder-7 (GAD-7) scale for anxiety. The PHQ-9 consists of nine items assessing the frequency of depressive symptoms over the past two weeks, with a total score of ≥10 indicating significant depressive symptoms [14]. Similarly, a GAD-7 score of ≥10 was considered indicative of moderate to severe anxiety [15]. Follow-up evaluations were performed at one and two years after baseline, including repeated measurements of monthly headache days, monthly severe headache days, and monthly medication days. Medication use at follow-up visits was also documented using the same classification of acute and preventive medications to allow for comparison of treatment exposure over time. At the one- and two-year follow-ups, medication overuse was reassessed according to the ICHD-3 criteria [11], defined as the use of ergotamine, triptans, opioids, or combination analgesics on ≥10 days per month or simple analgesics on ≥15 days per month. Medication overuse was considered resolved when patients no longer fulfilled the ICHD-3 criteria at follow-up.

### 2.3. Assessment of Interictal Burden

The MIBS-4 was administered at the two-year follow-up to assess the burden experienced by patients between headache attacks. This validated instrument measures various aspects of impairment, including difficulties at work or school, challenges in home and social life, difficulty making plans or appointments, and emotional and cognitive distress on migraine-free days [8,16]. The instrument has a 4-week recall period and consists of four items, each rated on a 4-point scale (0–3), where 0 = “never” or “do not know/not applicable”, 1 = “rarely”, 2 = “some of the time”, and 3 = “much of the time” or “most or all of the time.” The total score is calculated as the sum of the four items and ranges from 0 to 12. The MIBS-4 total score categorizes the interictal burden as follows: none (0), mild (1–2), substantial (3–4), and severe (≥5).

### 2.4. Ethics Statement

The initial and amended study protocols were approved by the Institutional Review Board of Hallym University Dongtan Sacred Heart Hospital. The initial protocol was approved on 25 March 2020 (IRB No. 2020-02-004), and the final amended protocol was approved on 4 October 2024 (IRB No. 2020-02-004-012). Written informed consent was obtained from all participants prior to study enrollment, in accordance with the Declaration of Helsinki.

### 2.5. Statistical Analysis

The normality of continuous variables was assessed using the Shapiro–Wilk test; as all continuous variables showed significant departure from normality (*p* < 0.05), they are presented as median [interquartile range (IQR)] throughout. Categorical variables are presented as frequencies and percentages. Participants were classified into two groups according to the presence of severe interictal burden (MIBS-4 ≥ 5 vs. <5). Group comparisons were performed using the Wilcoxon rank-sum test for continuous variables and the chi-squared test or Fisher’s exact test for categorical variables. Comparisons across the four MIBS-4 burden grades (none, mild, substantial, and severe) were performed using the Kruskal–Wallis test and the chi-squared test. Spearman rank correlation coefficients were used to evaluate the associations between MIBS-4 total scores and clinical variables. A two-tailed *p*-value of < 0.05 was considered statistically significant. All analyses were performed using R 4.5.2 (R Foundation for Statistical Computing, Vienna, Austria). This observational study was designed and reported in accordance with the STROBE guidelines. The completed STROBE checklist is provided as Table S6.

## 3. Results

### 3.1. Participants and Baseline Characteristics

A total of 149 patients with MOH were enrolled at baseline. The cohort was predominantly female (81.2%, *n* = 121), with a median age of 45.0 years [39.0–53.0] and a median body mass index of 22.9 kg/m^2^ [20.6–25.0]. The median duration of headache history was 23.0 years [17.0–31.0]. Baseline assessments indicated a substantial disease burden, with a median HIT-6 score of 66.0 [61.0–70.0] and a median MIDAS score of 47.0 [20.0–110.0]. Psychological assessments showed a median PHQ-9 score of 9.0 [6.0–13.0] and a median GAD-7 score of 5.0 [2.0–8.0] (Table 1).

### 3.2. One- and Two-Year Outcomes of Headache Characteristics

At the one-year follow-up, substantial improvements were observed across all headache-related outcomes, with monthly headache days decreasing from 20.0 [17.0–29.0] at baseline to 7.0 [4.0–10.0], monthly severe headache days from 10.0 [5.0–15.0] to 2.0 [0.0–5.0], and monthly medication days from 16.0 [13.0–23.0] to 5.0 [2.0–9.0]. The proportion of patients meeting the criteria for medication overuse decreased markedly from 100.0% at baseline to 24.2% at one year. These improvements were largely maintained at the two-year follow-up: monthly headache days were 6.0 [3.0–9.0], severe headache days were 2.0 [0.0–4.0] and medication days were 4.0 [2.0–7.0]. Meanwhile, the proportion of patients with medication overuse declined further to 17.1%, indicating sustained improvement in headache burden and medication overuse (Table 2).

Preventive medication use increased from 33.6% at baseline to 73.4% at one year and then declined to 35.0% at two years; the most prominent changes were observed for antiseizure medications (19.5% to 39.5% to 13.7%), onabotulinumtoxinA (4.7% to 21.8% to 3.4%), and calcitonin gene-related peptide (CGRP) monoclonal antibodies (2.0% to 26.6% to 12.0%) (Table S2).

Acute medication use remained high at one year and then decreased substantially by two years, with overall use changing from 97.3% at baseline to 100.0% at one year and 60.7% at two years. The most notable changes were a marked transient increase in triptan use from 43.0% to 92.7% at one year followed by a decline to 39.3% at two years, along with decreases in ergotamine (15.4% to 4.3%), simple analgesics (53.7% to 38.5%), and combination analgesics (24.8% to 1.7%) (Table S3). Two-year resolution of medication overuse did not differ significantly according to the baseline class of overused acute medication (Table S4). Resolution rates were 88.9% for ergotamine, 75.9% for triptans, 80.6% for simple analgesics, and 89.3% for combination analgesics (all *p* > 0.05, Fisher’s exact test). Triptan overuse showed a non-significant trend toward a lower likelihood of resolution (OR 0.39, *p* = 0.085). Opioid overuse was present in only one patient at baseline and was therefore not analyzed separately. Because the overused classes were not mutually exclusive, with 57 of 145 patients (39.3%) overusing two or more classes, each class was analyzed as use vs. non-use.

### 3.3. Interictal Burden at the Two-Year Follow-Up

Of the 149 patients, 117 (78.5%) had two-year MIBS-4 data available and were included in this analysis. There were no significant differences in age, sex, BMI, headache duration, or baseline monthly headache days between patients with and without two-year MIBS-4 data; however, those without two-year data had higher baseline monthly medication days, headache impact (HIT-6), and psychological symptom scores (PHQ-9 and GAD-7) (Table S1).

**Figure 2:**
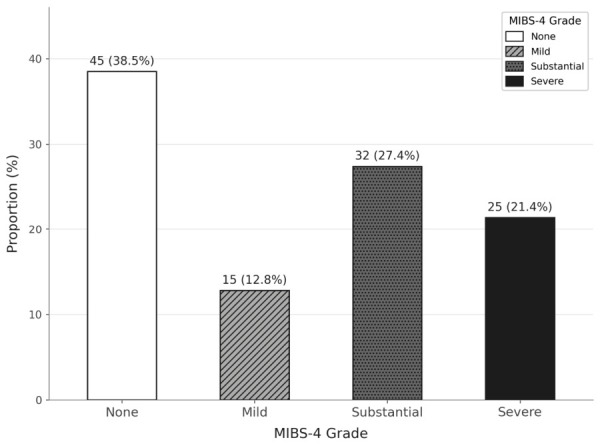
Distribution of MIBS-4 grades at the two-year follow-up. Distribution of Migraine Interictal Burden Scale (MIBS-4) grades among 117 patients with medication overuse headache (MOH) at two years. The proportions were as follows: no burden (38.5%), mild (12.8%), substantial (27.4%), and severe (21.4%). Substantial or severe burden persisted in 48.8% of patients. Abbreviations: MIBS-4, Migraine Interictal Burden Scale-4; MOH, medication overuse headache.

The median total MIBS-4 score was 2.0 [0.0–4.0]. Based on MIBS-4 grading, 45 patients (38.5%) reported no interictal burden, 15 (12.8%) mild burden, 32 (27.4%) substantial burden, and 25 (21.4%) severe burden. The distribution of MIBS-4 grades at the two-year follow-up is shown in Figure 2. Among the four MIBS-4 items, the highest median score was reported for worry about planning social or leisure activities (median, 1.0 [IQR, 0.0–2.0]), indicating that this was the most prominent residual burden (Table 2).

Compared with patients without severe interictal burden at two years, those with severe burden showed similar baseline demographic characteristics and similar baseline monthly headache days and monthly medication days, but had significantly greater headache-related impact and disability, with higher HIT-6 (68.0 vs. 64.0, *p* = 0.019) and MIDAS scores (110.0 vs. 36.0, *p* = 0.002). They also had greater psychological burden, with higher PHQ-9 (11.0 vs. 8.0, *p* = 0.032) and GAD-7 scores (7.0 vs. 4.0, *p* = 0.010), were more likely to be current smokers (20.0% vs. 4.3%, *p* = 0.036), and more frequently used preventive medications at baseline (48.0% vs. 25.0%, *p* = 0.048, Table 3). These findings represent univariate associations; given the limited number of patients with severe interictal burden, they should not be interpreted as independent predictors. Despite resolution of medication overuse, 14 patients (14.4%) continued to report severe interictal burden at two years. Although this proportion was markedly lower than that in patients with ongoing medication overuse (55.0%), the finding suggests that substantial interictal burden may be observed even after medication overuse has resolved (Table 4).

## 4. Discussion

To our knowledge, this is the first study to evaluate interictal burden in patients with MOH over a two-year follow-up period. The prospective cohort design, relatively large sample size, and use of standardized, validated instruments, including the MIBS-4, support the reliability and clinical relevance of our findings. Interictal burden remains a significant challenge in patients with MOH. In this study, a substantial proportion of patients continued to experience interictal burden two years after diagnosis, despite marked improvements in headache frequency and medication use. These findings suggest that even when conventional headache metrics improve, meaningful disability may be present during headache-free periods. Because interictal burden was assessed only at the two-year follow-up, these findings represent a cross-sectional snapshot rather than a measure of change from baseline; the interictal burden observed should therefore be interpreted as a residual state at two years rather than as evidence of improvement or worsening over time. Moreover, because this single-timepoint design cannot establish temporal or causal relationships, the factors associated with severe interictal burden should be regarded as correlates rather than causal determinants, and their direction of association cannot be inferred from the present data.

The concept of interictal burden has emerged as an important dimension in headache research, capturing impairment extending beyond the ictal phase. The MIBS-4 has been reported to correlate with well-being, anxiety sensitivity and worst pain severity experienced by migraine sufferers undergoing CGRP monoclonal antibody therapy [17]. Previous studies have shown that higher MIBS-4 scores are associated with reduced well-being, psychiatric comorbidity, and greater headache frequency, highlighting the clinical relevance of this measure [9,18]. Unlike traditional ictal metrics such as monthly headache days or medication use, interictal burden reflects the broader psychological and functional impact of migraines on daily life [19]. The rate of severe interictal burden in our cohort at two years (21.4%) was lower than that reported in cross-sectional studies of active migraine, possibly reflecting clinical improvement after detoxification and structured follow-up, although natural disease fluctuation cannot be excluded [18]. A Japanese study suggested that interictal burden may predict work productivity impairment more strongly than ictal burden, underscoring its clinical relevance [20]. From a clinical management perspective, the MIBS-4 ≥ 5 threshold may serve as a practical marker to identify patients warranting closer attention during follow-up. Although patients later classified as having severe interictal burden did not differ from the remainder in headache-related measures at baseline, at the two-year follow-up they had significantly higher monthly headache days, severe headache days, and monthly medication days than those below the threshold (Table S5), indicating that a MIBS-4 ≥ 5 score marks a subgroup with a less favorable clinical course rather than a group already distinguished by attack frequency at the outset. Consistent with this, prior work has shown that MIBS-4 scores are associated with headache-related disability (HIT-6) but not with monthly migraine days, supporting the scale’s value in capturing dimensions of burden not fully reflected by attack frequency alone [21]. Accordingly, routine application of the MIBS-4 may support more individualized management beyond headache-frequency control [22], with a score ≥5 prompting closer monitoring, assessment of comorbid anxiety and depression, and consideration of targeted preventive or psychological interventions.

The most prominent interictal domain in our cohort was worry about planning social or leisure activities, consistent with the existing literature identifying anticipatory anxiety as a core feature of the interictal experience [23,24]. Nonetheless, interictal burden remains underrecognized in routine practice, with surveys of Korean neurologists indicating underuse of validated assessment tools and structured monitoring [25].

Acute medication exposure declined substantially during follow-up. The proportion of patients using any acute medication decreased from 97.3% at baseline to 60.7% at two years, with marked reductions in combination analgesics and ergotamine. Notably, opioid use was uncommon in our cohort, with only one patient overusing opioids at baseline, and opioid use was no longer reported at two years; the low rate of opioid overuse should be considered when interpreting our favorable outcomes, as opioid use is generally associated with poorer prognosis in MOH. Accordingly, medication overuse decreased from 100% at baseline to 17.1% at two years. Preventive medication increased at 1 year (73.4%), and subsequently decreased to near-baseline levels (35.0%). Previous studies have shown that preventive therapy following withdrawal of overused medications can reduce headache frequency and medication use in MOH [26,27,28,29]. Consistent with these findings, our cohort also demonstrated improvements in headache frequency and medication use. The decline in preventive treatment use between the first- and second-year follow-up may reflect several factors. In routine clinical practice, preventive therapies are often tapered or discontinued after sustained improvement in headache frequency and resolution of medication overuse. In addition, reimbursement policies and out-of-pocket costs may influence the long-term continuation of treatments such as CGRP monoclonal antibodies and onabotulinumtoxinA. Because reasons for treatment discontinuation were not systematically collected, the relative contribution of clinical improvement, inadequate response, adverse events, financial considerations, or patient preference could not be determined. Previous real-world data from Korea have also demonstrated frequent treatment discontinuation and switching among patients with migraine, highlighting the complexity of long-term treatment adherence in clinical practice [30]. We further examined whether these outcomes differed by the class of overused medication. Previous studies have suggested that treatment outcomes in MOH may vary according to the class of overused medication [31], with opioid and barbiturate overuse generally associated with poorer outcomes and triptans or ergots with more favorable ones [32]. In our cohort, however, we did not observe significant differences in two-year resolution rates across medication classes. Notably, opioid overuse, which represents the most treatment-resistant end of this spectrum, was present in only one patient in our cohort, so this gradient could not be adequately tested. These findings should therefore be interpreted cautiously, given the substantial overlap between medication classes and the small size of several subgroups.

The long-term prognosis of MOH following resolution of medication overuse remains an area of ongoing investigation. Prior studies have reported that successful withdrawal is achievable in approximately 50–70% of patients at one year [28,33]; however, relapse rates reach 20–45% within the first year after withdrawal and rise to 40–50% over longer follow-up periods, with the majority of relapses occurring within the first 12 months after detoxification [34]. Identified predictors of relapse include longer duration of primary headache, greater baseline headache frequency, use of combination analgesics, and comorbid smoking and alcohol use [35]. Against this backdrop, the two-year medication overuse rate of 17.1% observed in our cohort is notably lower than relapse rates reported in many prior studies, suggesting a relatively favorable long-term outcome. This may partly reflect the structured follow-up and the availability of advanced preventive therapies, including CGRP monoclonal antibodies and onabotulinumtoxinA, during the follow-up period [36]. Nevertheless, the presence of substantial or severe interictal burden in nearly half of patients suggests that freedom from medication overuse does not equate to full functional recovery, and that long-term monitoring beyond relapse prevention is warranted [20,37]. These results, however, derive from a single-center sub-study of a multicenter registry, and the relatively favorable outcomes observed here may not be fully generalizable to other settings.

Notably, substantial interictal burden has been reported even among patients receiving preventive therapies, suggesting that reductions in headache frequency do not necessarily translate into full functional recovery [17,38,39]. In MOH, this distinction is particularly relevant, as the condition increasingly appears to overlap with chronic migraine biology rather than representing a purely reversible pharmacologic phenomenon [40]. Neuroimaging studies have demonstrated functional alterations in the pain processing network in patients with MOH, supporting persistent central sensitization even after withdrawal of overused medications [41,42]. Because most patients in this cohort had migraine as the underlying primary headache disorder, this overlap may partly explain the interictal burden observed during long-term follow-up. Among patients initiating CGRP monoclonal antibody therapy, data from a phase III randomized, placebo-controlled trial suggest that targeted preventive therapy is associated with significant reductions in MIBS-4 scores compared with placebo, including a marked decrease in severe interictal burden [9]. These findings suggest that preventive therapy may improve interictal outcomes alongside ictal metrics. Consistent with this perspective, the observation that 14.4% of patients with resolved medication overuse still reported severe interictal burden supports the notion that interictal burden represents a distinct dimension of disease not fully captured by headache frequency or acute medication use alone [9]. The impact of different therapeutic approaches on interictal outcomes should be interpreted cautiously. Preventive treatment increased substantially during the first year, including the use of onabotulinumtoxinA and CGRP monoclonal antibodies. However, treatments were not allocated in a controlled manner, and patients with greater baseline severity were more likely to receive preventive therapies. Accordingly, the observed association between baseline preventive treatment and severe interictal burden at two years most likely reflects confounding by indication rather than a treatment effect. Therefore, the differential impact of specific therapies on interictal burden cannot be determined from the present observational data and warrants prospective investigation. Despite substantial reductions in medication overuse and improvements in headache frequency, nearly half of patients continued to report substantial or severe interictal burden at two years. These findings suggest that reductions in headache frequency alone may be insufficient to restore full functional well-being in a considerable subset of patients. The association of current smoking with severe interictal burden was notably more prevalent in affected patients (20.0% vs. 4.3%, *p* = 0.036), consistent with prior evidence linking smoking to poorer headache outcomes and medication overuse relapses, although whether this reflects an independent risk factor or a marker of a broader high-risk psychological profile warrants further investigation.

Several limitations should be acknowledged. First, although the parent registry was multicenter, the present analysis was a single-center sub-study, which—together with loss to follow-up over the two-year period—may have introduced selection bias and limited the generalizability of the findings. In addition, recruitment from a single center may select for a particular referral population, which could partly contribute to the relatively favorable outcomes observed. Age, sex, headache duration, and baseline headache frequency were broadly comparable between patients with and without two-year MIBS-4 data; however, those lost to follow-up had higher baseline medication use, headache impact, and psychological symptom scores (Table S1), suggesting that some degree of attrition bias cannot be excluded and that the analyzed cohort may represent a comparatively less severe subgroup. Second, interictal burden was assessed only at the two-year follow-up using self-reported questionnaires, which precludes evaluation of temporal changes and may introduce recall bias; serial assessment of MIBS-4 scores from baseline through follow-up visits would be needed to determine whether interictal burden improves progressively or plateaus after initial treatment. This single-timepoint design also precludes causal inference, so the factors associated with severe interictal burden should be regarded as correlates rather than determinants. Third, although medication overuse status was reassessed according to the ICHD-3 criteria, formal diagnostic reclassification into alternative primary headache categories was not systematically performed; it is therefore possible that some patients may have transitioned to a diagnosis of chronic migraine without medication overuse, which may have influenced the interpretation of long-term outcomes. Because the underlying primary headache disorder was migraine in most patients, the interictal burden observed at two years may partly reflect ongoing chronic migraine rather than a residual feature of MOH; this distinction could not be disentangled in the present design and may affect both the interpretation of outcomes and the attribution of interictal burden. Fourth, given the limited number of patients with severe interictal burden (*n* = 25), a multivariable regression analysis adjusting for psychological variables (PHQ-9, GAD-7) and smoking status was not feasible without a substantial risk of overfitting and unstable estimates. The observed relationships with severe interictal burden should therefore be interpreted as univariate associations rather than independent predictors.

## 5. Conclusions

In this two-year follow-up study, severe interictal burden was observed in some patients with MOH despite improved headache frequency and medication use. Long-term management should extend beyond medication withdrawal, and the MIBS-4 may be a useful tool to monitor interictal impairment.

## Figures and Tables

**Table 1:** Baseline clinical characteristics of patients with MOH (*N* = 149).

Variable	Level	Overall
Age, years (median [IQR])		45.0 [39.0, 53.0]
Female sex (%)		121 (81.2)
BMI, kg/m^2^ (median [IQR])		22.9 [20.6, 25.0]
Duration of headache onset, years (median [IQR])		23.0 [17.0, 31.0]
Smoking status (%)		
Never		136 (91.3)
Ex-smoker		1 (0.7)
Current		12 (8.1)
Caffeine intake (%)		
No		45 (30.2)
Low (1–200 mg/d)		69 (46.3)
High (>200 mg/d)		35 (23.5)
Alcohol use (%)		20 (13.4)
Monthly headache days (median [IQR])		20.0 [17.0, 29.0]
Monthly severe headache days (median [IQR])		10.0 [5.0, 15.0]
Monthly medication days (median [IQR])		16.0 [13.0, 23.0]
HIT-6 score (median [IQR])		66.0 [61.0, 70.0]
MIDAS score (median [IQR])		47.0 [20.0, 110.0]
PHQ-9 score (median [IQR])		9.0 [6.0, 13.0]
GAD-7 score (median [IQR])		5.0 [2.0, 8.0]
Preventive treatment at baseline (%)	Yes	50 (33.6)
Acute medication use at baseline (%)	Yes	145 (97.3)

Continuous variables are presented as median [IQR] (Shapiro–Wilk *p* < 0.05 for all). Categorical variables are presented as n (%). MOH, Medication overuse headache; BMI, Body mass index; HIT-6, Headache impact test-6; MIDAS, Migraine disability assessment; PHQ-9, Patient health questionnaire-9; GAD-7, General anxiety disorder-7.

**Table 2:** Clinical outcomes at baseline, 1-year, and 2-year follow-up after MOH diagnosis.

Characteristics	Baseline	1-Year FU	2-Year FU
*N*	149	124	117
Headache parameters			
Monthly headache days	20.0 [17.0, 29.0]	7.0 [4.0, 10.0]	6.0 [3.0, 9.0]
Monthly severe headache days	10.0 [5.0, 15.0]	2.0 [0.0, 5.0]	2.0 [0.0, 4.0]
Monthly medication days	16.0 [13.0, 23.0]	5.0 [2.0, 9.0]	4.0 [2.0, 7.0]
Medication overuse, n (%) †	149 (100.0)	30 (24.2)	20 (17.1)
*N*			117
MIBS-4 (past 4 weeks)			
Q1: Impact on work/school			0.0 [0.0, 1.0]
Q2: Worry about planning social/leisure activities			1.0 [0.0, 2.0]
Q3: Impact on life			0.0 [0.0, 1.0]
Q4: Feeling helpless			0.0 [0.0, 1.0]
MIBS-4 total score			2.0 [0.0, 4.0]
MIBS-4 grade, n (%)			
None (0)			45 (38.5)
Mild (1–2)			15 (12.8)
Substantial (3–4)			32 (27.4)
Severe (≥5)			25 (21.4)

Continuous variables are presented as median [IQR] (Shapiro–Wilk *p* < 0.05 for all). † Medication overuse: triptans or combination drugs ≥ 10 days/month or simple analgesics ≥ 15 days/month. MOH, Medication overuse headache; FU, Follow-up.

**Table 3:** Comparison of baseline features of patients by severe interictal burden at 2-year follow-up (MIBS-4 ≥ 5).

Variable	No (*n* = 92)	Yes (*n* = 25)	*p*-Value
Age, years (median [IQR])	44.0 [39.0, 54.0]	46.0 [42.0, 53.0]	0.315
Female sex (%)	78 (84.8)	21 (84.0)	1.000
BMI, kg/m^2^ (median [IQR])	22.8 [20.3, 24.9]	23.4 [20.8, 25.5]	0.485
Duration of headache onset, years (median [IQR])	23.0 [17.0, 30.3]	28.0 [19.0, 36.0]	0.156
Smoking status (%)			0.036
Never	87 (94.6)	20 (80.0)	
Ex-smoker	1 (1.1)	0 (0.0)	
Current	4 (4.3)	5 (20.0)	
Caffeine intake (%)			0.127
No	30 (32.6)	3 (12.0)	
Low (1–200 mg/d)	42 (45.7)	15 (60.0)	
High (>200 mg/d)	20 (21.7)	7 (28.0)	
Alcohol use (%)	14 (15.2)	3 (12.0)	1.000
Monthly headache days (median [IQR])	20.0 [16.8, 28.0]	21.0 [20.0, 25.0]	0.684
Monthly severe headache days (median [IQR])	10.0 [5.0, 15.0]	7.0 [5.0, 10.0]	0.282
Monthly medication days (median [IQR])	15.0 [12.0, 20.0]	20.0 [12.0, 23.0]	0.456
HIT-6 score (median [IQR])	64.0 [60.0, 68.0]	68.0 [63.0, 71.0]	0.019
MIDAS score (median [IQR])	36.0 [15.0, 90.0]	110.0 [45.0, 165.0]	0.002
PHQ-9 score (median [IQR])	8.0 [5.0, 12.0]	11.0 [8.0, 14.0]	0.032
GAD-7 score (median [IQR])	4.0 [1.5, 8.0]	7.0 [5.0, 12.0]	0.010
Preventive treatment at baseline (%)	23 (25.0)	12 (48.0)	0.048

Continuous variables are presented as median [IQR] (Shapiro–Wilk *p* < 0.05 for all). *p*-value by Wilcoxon rank-sum test for continuous variables/Chi-squared or Fisher’s exact test for categorical variables. BMI, Body mass index; HIT-6, Headache impact test-6; MIDAS, Migraine disability assessment; PHQ-9, Patient health questionnaire-9; GAD-7, General anxiety disorder-7.

**Table 4:** Interictal burden by medication overuse status at 2-year follow-up.

Group	N	MIBS-4 Total	Severe Burden, n (%)	MHD (2 yr)	MedD (2 yr)
MO ongoing	20	5.0 [1.8, 9.2]	11 (55.0)	15.0 [15.0, 20.0]	15.0 [10.0, 20.0]
MO resolved	97	1.0 [0.0, 4.0]	14 (14.4)	4.0 [3.0, 7.0]	3.0 [1.0, 5.0]
*p* value		<0.001	<0.001	<0.001	<0.001

Medication overuse (MO): triptans or combination drugs ≥ 10 days/month or simple analgesics ≥ 15 days/month. *p*-value by Wilcoxon rank-sum test (continuous)/Fisher’s exact test (categorical). MO, Medication overuse; MHD, Monthly headache days; MedD, Monthly medication days.

## Data Availability

The datasets used and/or analyzed during the current study are available from the corresponding author on reasonable request.

## Supplementary Materials

The following supporting information can be downloaded at https://www.mdpi.com/article/10.3390/jcm15124785/s1. Table S1: Comparison of baseline characteristics between patients with and without two-year MIBS-4 data; Table S2: Preventive medication use at baseline, one-year, and two-year follow-up; Table S3: Acute medication use at baseline, one-year, and two-year follow-up; Table S4: Two-year resolution of medication overuse stratified by baseline class of overused acute medication; Table S5: Headache parameters at the two-year follow-up by severe interictal burden (MIBS-4 ≥ 5); Table S6: Completed STROBE checklist.

## Abbreviations

MOH	Medication overuse headache
MIBS-4	Migraine Interictal Burden Scale-4
ICHD-3	International Classification of Headache Disorders, 3rd edition
HIT-6	Headache Impact Test-6
MIDAS	Migraine Disability Assessment Scale
PHQ-9	Patient Health Questionnaire-9
GAD-7	Generalized Anxiety Disorder-7
IRB	Institutional Review Board
IQR	Interquartile range
CGRP	Calcitonin gene-related peptide
