# Supplementary material for: Two-Year Outcomes and Interictal Burden After Treatment for Medication Overuse Headache
Source: J Clin Med. 2026 Jun 19;15(12):4785. doi: 10.3390/jcm15124785 (PMC13302636; doi:10.3390/jcm15124785)
Supplement: Supplementary file 1 [file jcm-15-04785-s001.zip › jcm-4329029-supplementary.pdf]

Supplementary Table S1. Comparison of baseline characteristics between patients with and without two-year MIBS-4 data

**Variable**	**MIBS-4 data available**	**No**	**Yes**	**p**	**sig**
**n**		**32**	**117**		
Age, years (median [IQR])		44.00 [33.75, 50.25]	45.00 [39.00, 54.00]	0.248	
Female sex (%)	No	10 (31.2)	18 (15.4)	0.075	
	Yes	22 (68.8)	99 (84.6)		
BMI, kg/m² (median [IQR])		23.00 [20.88, 24.95]	22.80 [20.30, 25.00]	0.455	
Duration of headache onset, years (median [IQR])		20.00 [13.75, 24.75]	24.00 [17.00, 32.00]	0.133	
Monthly headache days (median [IQR])		27.50 [19.25, 30.00]	20.00 [17.00, 28.00]	0.074	
Monthly severe headache days (median [IQR])		10.00 [5.00, 15.00]	10.00 [5.00, 15.00]	0.751	
Monthly medication days (median [IQR])		20.00 [15.00, 28.25]	16.00 [12.00, 20.00]	0.044	**
HIT-6 score (median [IQR])		68.00 [65.00, 72.50]	65.00 [61.00, 70.00]	0.006	**
MIDAS score (median [IQR])		57.50 [30.00, 101.25]	45.00 [20.00, 114.00]	0.517	
PHQ-9 score (median [IQR])		11.00 [7.75, 14.25]	8.00 [5.00, 12.00]	0.029	**
GAD-7 score (median [IQR])		7.00 [5.00, 11.00]	5.00 [2.00, 8.00]	0.026	**
Preventive treatment at baseline (%)	No	17 (53.1)	82 (70.1)	0.112	
	Yes	15 (46.9)	35 (29.9)		

Continuous variables are presented as median [IQR] (Shapiro-Wilk P < 0.05 for all).

P by Wilcoxon rank-sum test for continuous variables / Chi-squared or Fisher's exact test for categorical variables.

Supplementary Table S2. Preventive medication use at baseline, one-year, and two-year follow-up.

**Medication Category**	**Baseline (N=149)**	**1-year FU (N=124)**	**2-year FU (N=117)**
Any preventive drug use, (%)	50 (33.6)	91 (73.4)	41 (35.0)
TCA, (%)	21 (14.1)	23 (18.5)	12 (10.3)
Beta-blockers, (%)	14 (9.4)	14 (11.3)	9 (7.7)
Calcium channel blockers, (%)	6 (4.0)	4 (3.2)	3 (2.6)
Antiseizure medications, (%)	29 (19.5)	49 (39.5)	16 (13.7)
OnabotulinumtoxinA (Botox), (%)	7 (4.7)	27 (21.8)	4 (3.4)
Angiotensin receptor blocker, (%)	5 (3.4)	13 (10.5)	5 (4.3)
GONB/TNB, (%)	1 (0.7)	12 (9.7)	4 (3.4)
CGRP monoclonal antibody, (%)	3 (2.0)	33 (26.6)	14 (12.0)
Others, (%)	3 (2.0)	3 (2.4)	6 (5.1)

Data are presented as number (%).

TCA, tricyclic antidepressant; GONB, greater occipital nerve block; TNB, trigeminal nerve block; CGRP, calcitonin gene-related peptide; FU, follow-up.

Supplementary Table S3. Acute medication use at baseline, one-year, and two-year follow-up.

Medication Category	Baseline (N=149)	1-year FU (N=124)	2-year FU (N=117)
Any acute medication use, (%)	145 (97.3)	124 (100.0)	71 (60.7)
Ergotamine, (%)	23 (15.4)	5 (4.0)	5 (4.3)
Triptans, (%)	64 (43.0)	115 (92.7)	46 (39.3)
Simple analgesics, (%) (NSAIDs or acetaminophen)	80 (53.7)	90 (72.6)	45 (38.5)
Opioids, (%)	1 (0.7)	1 (0.8)	0 (0.0)
Combination analgesics, (%)	37 (24.8)	3 (2.4)	2 (1.7)
Others, (%)	1 (0.7)	0 (0.0)	2 (1.7)

Data are presented as number (%).

NSAIDs, nonsteroidal anti-inflammatory drugs; FU, follow-up.

Supplementary Table S4. Two-year resolution of medication overuse stratified by baseline class of overused acute medication.

Baseline overused class	Patients assessed at 2-year FU, n	Medication overuse resolved, n (%)	*p*-value
Ergotamine	18	16 (88.9)	0.734
Triptan	54	41 (75.9)	0.085
Simple analgesic	62	50 (80.6)	0.624
Combination analgesic	28	25 (89.3)	0.396
All patients	117	97 (82.9)	

Data are presented as number (%). Medication overuse was considered resolved when absent at the 2-year follow-up.

Each class was analyzed as use versus non-use because the overused classes were not mutually exclusive; 57 of 145 patients overused ≥2 classes at baseline.

**p*-value by Fisher's exact test comparing patients who did versus did not overuse medication class. Opioid overuse was present in only one patient at baseline and was therefore not analyzed separately.

Supplementary Table S5. Headache parameters at the two-year follow-up by severe interictal burden (MIBS-4 ≥ 5)

**Variable**	**No (n =92)**	**Yes (n =25)**	***p*-value**
Monthly headache days, median [IQR]	4.0 [4.0, 7.0]	10.0 [6.0, 15.0]	< 0.001
Monthly severe headache days, median [IQR]	1.0 [0.0, 3.0]	5.0 [2.0, 10.0]	0.001
Monthly medication days, median [IQR]	3.0 [1.0, 6.0]	7.0 [5.0, 15.0]	< 0.001

Continuous variables are presented as median [IQR] (Shapiro-Wilk P < 0.05 for all).

* p-value by Wilcoxon rank-sum (Mann-Whitney U) test. Groups are defined by interictal burden severity at the 2-year follow-up (MIBS-4 ≥ 5 vs. < 5).

Supplementary Table S6. Completed STROBE checklist

STROBE Statement—checklist of items that should be included in reports of observational studies

	Item No.	Recommendation	Page No.	Relevant text from manuscript
**Title and abstract**	1	(*a*) Indicate the study's design with a commonly used term in the title or the abstract	Page 1	"This study was derived from a prospective multicenter cohort of patients with MOH, using data from a single center."
		(*b*) Provide in the abstract an informative and balanced summary of what was done and what was found	Page 1	"Of 149 patients enrolled between April 2020 and November 2022, 117 (78.5%) completed the two-year follow-up… Among 117 patients…, 25 (21.4%) had severe interictal burden…"
**Introduction**				
Background/rationale	2	Explain the scientific background and rationale for the investigation being reported	Page 2	"few studies have examined long-term outcomes in MOH, and those that have are typically restricted to follow-up periods of 6 to 12 months. As a result, little is known about the trajectory of interictal burden in MOH…"
Objectives	3	State specific objectives, including any prespecified hypotheses	Page 2	"In this single-center sub-study of a prospective MOH registry, we evaluated interictal burden at two years using the validated MIBS-4 ~ identifying its potential correlates."
**Methods**				
Study design	4	Present key elements of study design early in the paper	Page 2	"This study was based on the Registry for Load and Management of MEdicAtion

				OveruSE Headache, ~multicenter prospective observational study… the present analysis was conducted as a single-center sub-study of the multicenter registry."
Setting	5	Describe the setting, locations, and relevant dates, including periods of recruitment, exposure, follow-up, and data collection	Page 2	"149 patients with MOH were consecutively enrolled at a single headache center between April 2020 and November 2022… patients at this center underwent an extended two-year follow-up."
Participants	6	(*a*) *Cohort study*—Give the eligibility criteria, and the sources and methods of selection of participants. Describe methods of follow-up<br>*Case-control study*—Give the eligibility criteria, and the sources and methods of case ascertainment and control selection. Give the rationale for the choice of cases and controls<br>*Cross-sectional study*—Give the eligibility criteria, and the sources and methods of selection of participants	Page 2	"Eligible participants were required to be aged ≥19 years, able to communicate and complete study questionnaires, and to provide written informed consent. Patients were excluded if they had severe neurological, psychiatric, or systemic medical conditions…"
		(*b*) *Cohort study*—For matched studies, give matching criteria and number of exposed and unexposed<br>*Case-control study*—For matched studies, give matching criteria and the number of controls per case	N/A	
Variables	7	Clearly define all outcomes, exposures, predictors, potential confounders, and effect modifiers. Give diagnostic criteria, if applicable	Page 2,4	"MOH according to ICHD-3… medication overuse, defined as ergotamine, triptans, opioids, or combination analgesics on ≥10 days/month, or simple analgesics on ≥15 days/month." + "MIBS-4… scores ≥5 indicating severe burden.”

Data sources/ measurement	8*	For each variable of interest, give sources of data and details of methods of assessment (measurement). Describe comparability of assessment methods if there is more than one group	Page 4	“Headache-related disability was measured using the Migraine Disability Assessment Scale (MIDAS) [13]. Psychological comorbidities were evaluated using the Patient Health Questionnaire-9 (PHQ-9) for depression and the Generalized Anxiety Disorder-7 (GAD-7) scale for anxiety.” “The MIBS-4 was administered at the two-year follow-up to assess the burden experienced by patients between headache attacks.”
Bias	9	Describe any efforts to address potential sources of bias	Page 12 Table S1	"the present analysis was a single-center sub-study, which—together with loss to follow-up… may have introduced selection bias… those lost to follow-up had higher baseline medication use, headache impact, and psychological symptom scores (Table S1), suggesting that some degree of attrition bias cannot be excluded…"
Study size	10	Explain how the study size was arrived at	Page 2,3 Figure 1	"Of the 149 patients enrolled at baseline, 124 completed the

	one-year follow-up… and 117 patients (78.5%) completed the full two-year follow-up and were included in the final analysis (Figure 1)."

Continued on next page

Quantitative variables	11	Explain how quantitative variables were handled in the analyses. If applicable, describe which groupings were chosen and why	Page 5	"continuous variables… are presented as median [IQR]… Participants were classified into two groups according to the presence of severe interictal burden (MIBS-4 ≥ 5 vs. < 5)… Comparisons across the four MIBS-4 burden grades (none, mild, substantial, severe)…"
Statistical methods	12	(*a*) Describe all statistical methods, including those used to control for confounding	Page 5	"Group comparisons were performed using the Wilcoxon rank-sum test… and the chi-squared test or Fisher's exact test… Spearman rank correlation… A two-tailed p < 0.05… All analyses were performed using R 4.5.2."
		(*b*) Describe any methods used to examine subgroups and interactions	N/A	
		(*c*) Explain how missing data were addressed	N/A	
		(*d*) *Cohort study*—If applicable, explain how loss to follow-up was addressed<br>*Case-control study*—If applicable, explain how matching of cases and controls was addressed<br>*Cross-sectional study*—If applicable, describe analytical methods taking account of sampling strategy	Page 7, Table S1	"those without two-year data had higher baseline monthly medication days, headache impact (HIT-6), and psychological symptom scores (PHQ-9 and GAD-7) (Table S1)."
		(*e*) Describe any sensitivity analyses	N/A	
**Results**				
Participants	13*	(a) Report numbers of individuals at each stage of study—eg numbers potentially eligible, examined for eligibility, confirmed eligible, included in the study, completing follow-up, and analysed	Page 2,3 Figure 1	"Of the 149 patients enrolled at baseline, 124 completed the one-year follow-up (25 lost to follow-up), and 117 patients (78.5%)

				completed the full two-year follow-up."
		(b) Give reasons for non-participation at each stage	Page 3, Figure 1	25 lost to follow-up" (1 년) / "7 additional patients lost to follow-up" (2 년) — Figure 1
		(c) Consider use of a flow diagram	Page 3, Figure 1	"Figure 1. Patient selection and follow-up"
Descriptive data	14*	(a) Give characteristics of study participants (eg demographic, clinical, social) and information on exposures and potential confounders	Page 5,6 Table 1	"predominantly female (81.2%, n = 121), median age 45.0 years [39.0–53.0], BMI 22.9 kg/m²… HIT-6 66.0… MIDAS 47.0… PHQ-9 9.0… GAD-7 5.0 (Table 1)."
		(b) Indicate number of participants with missing data for each variable of interest	N/A	
		(c) *Cohort study*—Summarise follow-up time (eg, average and total amount)	N/A	
Outcome data	15*	*Cohort study*—Report numbers of outcome events or summary measures over time	Page 6,7 Table 2	"Medication overuse decreased from 100% at baseline to 24.2% at one year and 17.1% at two years… (Table 2)."
		Case-control study—Report numbers in each exposure category, or summary measures of exposure	N/A	
		Cross-sectional study—Report numbers of outcome events or summary measures	N/A	
Main results	16	(*a*) Give unadjusted estimates and, if applicable, confounder-adjusted estimates and their precision (eg, 95% confidence interval). Make clear which confounders were adjusted for and why they were included	Page 8, 9 Table 3,4	Table 3·4. "higher HIT-6 (68.0 vs. 64.0, p = 0.019) and MIDAS (110.0 vs. 36.0, p = 0.002)… These findings represent univariate associations…" + "14.4% of patients with resolved medication overuse still reported severe interictal burden (Table 4)."

	(*b*) Report category boundaries when continuous variables were categorized	Page 4	"MIBS-4… none (0), mild (1–2), substantial (3–4), and severe (≥5)."
	(*c*) If relevant, consider translating estimates of relative risk into absolute risk for a meaningful time period	N/A	

Continued on next page

Other analyses	17	Report other analyses done—eg analyses of subgroups and interactions, and sensitivity analyses	Page 9,<br>Table 4,<br>Table S4,S5	
**Discussion**				
Key results	18	Summarise key results with reference to study objectives	Page 9	"To our knowledge, this is the first study to evaluate interictal burden in patients with MOH over a two-year follow-up period… a substantial proportion of patients continued to experience interictal burden two years after diagnosis…"
Limitations	19	Discuss limitations of the study, taking into account sources of potential bias or imprecision. Discuss both direction and magnitude of any potential bias	Page 12	"Several limitations should be acknowledged. First… single-center sub-study, which—together with loss to follow-up… may have introduced selection bias… attrition bias cannot be excluded…"
Interpretation	20	Give a cautious overall interpretation of results considering objectives, limitations, multiplicity of analyses, results from similar studies, and other relevant evidence	Page 12	"reductions in headache frequency alone may be insufficient to restore full functional well-being… factors associated with severe interictal burden should be regarded as correlates rather than causal determinants."
Generalisability	21	Discuss the generalisability (external validity) of the study results	Page 11	"these results… derive from a single-center sub-study of a multicenter registry, and the relatively favorable outcomes

				observed here may not be fully generalizable to other settings."
**Other information**				
Funding	22	Give the source of funding and the role of the funders for the present study and, if applicable, for the original study on which the present article is based	Page 13	"This research was funded by the Hallym University Research Fund 2025 (grant number HURF-2025-16)."

*Give information separately for cases and controls in case-control studies and, if applicable, for exposed and unexposed groups in cohort and cross-sectional studies.

Note: An Explanation and Elaboration article discusses each checklist item and gives methodological background and published examples of transparent reporting. The STROBE checklist is best used in conjunction with this article (freely available on the Web sites of PLoS Medicine at http://www.plosmedicine.org/, Annals of Internal Medicine at http://www.annals.org/, and Epidemiology at http://www.epidem.com/). Information on the STROBE Initiative is available at www.strobe-statement.org.
